# High-throughput assessment of *FMR1* and *SNRPN* methylation-based newborn screening using IsoPure and QIAcube HT systems

**DOI:** 10.1080/17501911.2025.2544530

**Published:** 2025-08-13

**Authors:** Caleb Cartagena, Mohammed Alshawsh, Minh Q. Bui, Dinusha Gamage, Rajvi P. Thakor, James Pitt, Ronda F. Greaves, Meg Wall, Richard Saffery, David J. Amor, David E. Godler

**Affiliations:** a Murdoch Children’s Research Institute, Royal Children’s Hospital, Parkville, VIC, Australia; b Department of Paediatrics, University of Melbourne, Parkville, VIC, Australia; c Department of Paediatrics, Monash University, Clayton, VIC, Australia; d Centre for Epidemiology & Biostatistics, Melbourne School of Population & Global Health, University of Melbourne, Carlton, VIC, Australia; e Victorian Clinical Genetics Services, Murdoch Children’s Research Institute, Parkville, VIC, Australia

**Keywords:** DNA methylation, high-throughput, bisulfite conversion, *FMR1*, *SNRPN*, fragile X syndrome, Prader-Willi syndrome, Angelman syndrome

## Abstract

**Aim:**

This study compared methylation-specific quantitative melt analysis of FMR1 and SNRPN methylation (mDNA) using automated bisulfite conversion by the magnetic-bead-based IsoPure and column-based QIAcube HT systems.

**Methods:**

Two bisulfite conversion methods were assessed on 3.2 mm punches from the same archival blood spots stored at room temperature for >10 years of individuals with FMR1 premutation (*n* = 20), fragile X syndrome (FXS, *n* = 20), or chromosome 15 imprinting disorders (*n* = 50) and freshly made blood spots from 184 newborns from the general population. Performance criteria were: (i) diagnostic sensitivity and specificity for the conditions screened; (ii) reaction failure rate; (iii) variability in mDNA between groups.

**Results:**

Both methods showed 100% sensitivity and specificity for differentiating FXS and individual chromosome 15 imprinting disorders. IsoPure showed reaction failure rates of 0.365% for SNRPN and 0.74% for FMR1 compared to 19.34% and 2.56%, for QIAcube HT, respectively, with most failed reactions originating from archival blood spots. IsoPure showed lower variability in mDNA values in the neurotypical and condition-specific ranges.

**Conclusion:**

The IsoPure system showed superior performance especially on archival samples, with broader applications for screening and diagnostic testing requiring high-throughput mDNA analyses on materials of limited quantity and quality.

## Plain Language Summary

Testing for a change to DNA known as methylation has been used by researchers and in medical practice to identify people affected with different diseases. We compared how well two procedures that employ robotics, work in a lab. We tested a small amount of blood soaked onto absorbent cards to detect this change. Blood on these cards was from people who either had fragile X syndrome or a chromosome 15 imprinting disorder or did not have these. Both procedures worked equally well on freshly made materials in identifying these conditions without making any mistakes. One of the procedures, however, worked better, on cards not recently made. These results provide new opportunities for automated testing of DNA to detect diseases.

## 1. Introduction

DNA methylation (mDNA) is involved in epigenetic regulation of the transcriptome [1–3]. Analysis of mDNA can be performed indirectly using bisulfite-converted DNA, a process with a large number of applications in diverse fields including gene regulation studies, disease diagnosis, monitoring, and personalized medicine [4,5]. We have previously used indirect mDNA analyses for diagnostic testing and screening applications to detect rare diseases including fragile X syndrome (FXS) and chromosome 15 imprinting disorders [6–8]. FXS is the most prevalent monogenic cause of inherited intellectual disability in males, caused by a CGG expansion in the 5’ UTR of the *FMR1* gene to ≥200 repeats, referred to as a full mutation (FM) [8–11]. FM leads to the mDNA of the *FMR1* promoter and the silencing of *FMR1*, resulting in the loss of its protein product, FMRP, which is essential for normal neurodevelopment [12]. Both CGG sizing and mDNA analysis of *FMR1* are used for standard-of-care diagnostic testing for FXS [8]. Chromosome 15 imprinting disorders, including Angelman (AS), Prader-Willi (PWS), and chromosome 15 duplication (Dup15q) syndromes, are caused by structural genetic mutations or epimutations within the imprinted region 15q11-q13[13,14]. mDNA analysis of *SNRPN* located in this region is also used for standard-of-care diagnostic testing for these conditions [14–16].

We have previously developed and applied methylation-specific quantitative melt-analysis(MS-QMA)test of *FMR1* and *SNRPN* promoters for diagnosis and screening applications [7,17–19]. For chromosome 15 imprinting disorders, we demonstrated its utility on newborn blood spots (NBS) from over 16,500 infants from the general population and have also applied the technique to dried blood spots (DBS) and high-quality DNA extracted from blood, saliva, and buccal epithelial cells from individuals affected by PWS, AS, and Dup15q syndrome [7,17–19]. Building on this, we are now applying MS-QMA as the first tier screening test in the Epi-Genomic Newborn screening (EpiGNs) program, to screen 100,000 infants from Victoria, Australia, for several rare genetic conditions, including FXS and chromosome 15 imprinting disorders [20]. The EpiGNs’ workflow was designed to meet existing requirements for newborn screening, utilizing a single 3.2 mm punch from an NBS for first-tier automated MS-QMA screening and an additional 3.2 mm punch to confirm etiology via epigenetic and genomic testing [20]. Our previous studies utilized the column-based QIAcube HT system (Qiagen, Hilden, Germany) for automated bisulfite conversion for indirect mDNA analysis.

This study examined performance of MS-QMA analysis of *FMR1* and *SNRPN* mDNA using automated bisulfite conversion by column-based QIAcube HT and magnetic bead-based IsoPure systems. We identified several potential advantages of the IsoPure compared to QIAcube HT-based bisulfite conversion including: (i) decreased use of plasticware with no tips required for the IsoPure system (resulting in significant cost saving); (ii) double the throughput for bisulfite conversion; (iii) requirement of a fume-hood with external suction for the Qiacube HT system due to aerosols generated by vacuum based washes of columns (not the case with magnetic bead based conversions). The primary objective of this study was therefore to determine if screening performance parameters could also be improved through use of the IsoPure magnetic bead-method for bisulfite conversion on the limited blood spot material available.

## 2. Materials and methods

### 2.1. Participants

The study was performed using dried blood samples from 20 participants with a *FMR1* premutation (PM: 55 to 199 CGG repeats, typically unmethylated and does not cause FXS) (50% female), 20 with an FM affected with FXS (55% female), 19 affected with PWS (63% female; 8 with deletion; 8 with maternal uniparental disomy [UPD]); 2 with imprinting center defect), 20 with AS (40% female; 8 deletions; 4 paternal UPD; 1 imprinting center defect, 6 with *UBE3A* sequence mutations, 1 with *SNRPN* mDNA mosaicism), 10 affected with maternal Dup15q syndrome [mDup15q] (40% female; 3 interstitial, 6 isodicentric, 1 tricentric) and one male affected with paternal Dup15q syndrome (pDup15q) (Supplementary Table S1). Participants were aged between 2 months and 45 years and were recruited as part of previous studies [7,21,22]. Control NBS samples used in this study were from 184 newborns (48% female) consented to provide blood spots for de-identified research at the Victorian Clinical Genetics Services (VCGS) between 2023 and 2024. All study procedures were approved by the Royal Children’s Hospital Human Research Ethics Committee (reference number: HREC/13/RCHM/24[v36] and HREC/92777/RCHM-2023[v2])

### 2.2. Sample processing

All NBS used were prospectively punched to provide two 3.2 mm punches per participant from materials remaining 2 weeks after the completion of standard newborn screening laboratory testing [20]. The NBS punches were stored at room temperature for less than 6 months before mDNA analysis. All DBS samples used were collected at the time of recruitment in the FREE FX study [22]. DBS samples were prepared by depositing 50 µL of venous blood onto a single disk of a Whatman 903 card and stored at room temperature for a period ranging from 3 to 11 years, as previously described [22].

**Figure 1.**
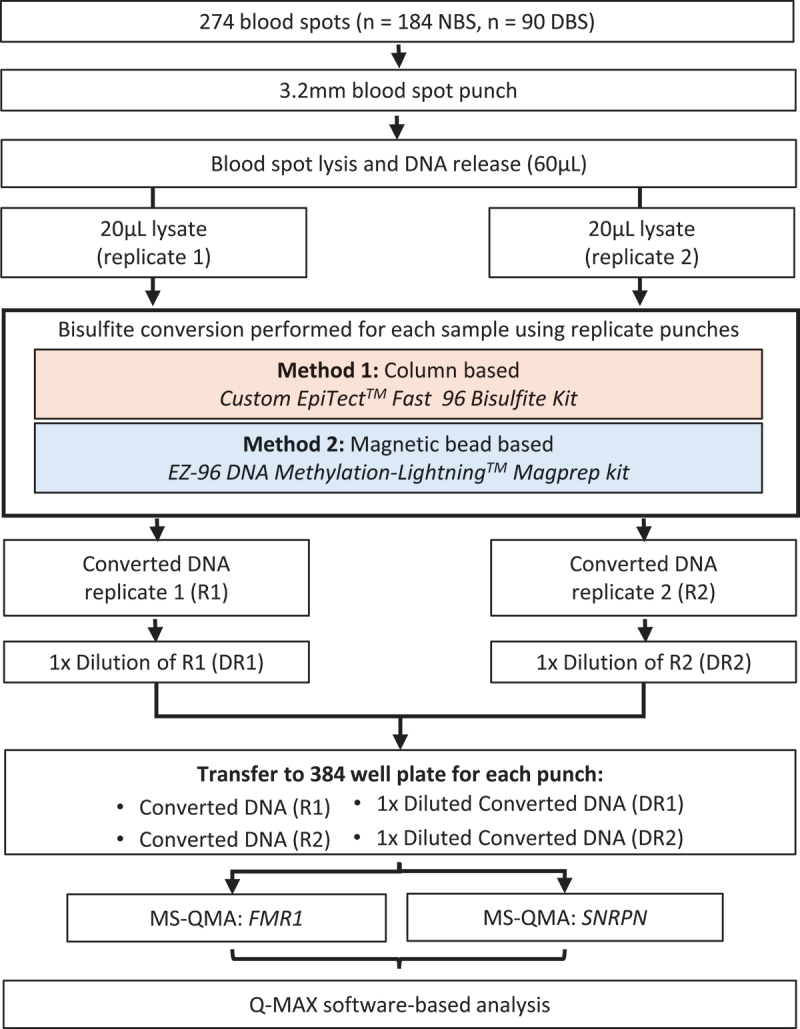
Overview of sample preparation and methylation analysis workflow. Sample preparation process and analysis for each newborn blood spot (NBS) and dried blood spot (DBS) sample used in the study. A single 3.2 mm punch from each sample was utilized for each bisulfite conversion method before undergoing methylation specific-quantitative analysis (MS-QMA) of *FMR1* and *SNRPN* promoters.

NBS and DBS samples were punched into replicate 96-well barcoded plates at 92 punches per plate using the automated Panthera-Puncher^TM^ 9 Instrument (Perkin Elmer, Connecticut, USA). Blood spots were then lysed in 60 µL of HS-buffer (Blue Life Solutions, Nevada, USA), aliquoted using the QIAcube HT system (Qiagen, Hilden, Germany) and subjected to temperature-controlled vortex mixing using the MultiTherm Shaker Heat and Cool (MultiTherm LLC, Pennsylvania, USA). For every plate of 92 lysed blood spot punches, 20 µL of blood spot lysates were transferred into two 96-well plates and stored at −30°C prior to bisulfite conversion (Figure 1).

### 2.3. mDNA analysis using the QIAcube HT and IsoPure systems

The first blood spot plate was bisulfite converted in replicate with EpiTect Fast 96 Bisulfite kits (Qiagen, Hilden, Germany) using the QIAcube HT Benchtop automation system (Qiagen, Hilden, Germany) as previously [7] (Supplementary Notes S1 and Figure S1). The second blood spot plate was bisulfite converted in replicate using the EZ-96 DNA Methylation-Lightning^TM^ Magprep kit (Zymo Research, California, USA) and the IsoPure™ 96 system (Accuris Instruments, Rhode Island, USA) as per manufacturer’s instructions (Zymo Research, California, USA) (Supplementary Notes S2 and Figures S2). Converted DNA in each of the two replicate plates (processed with either QIAcube HT or IsoPure systems) was diluted 1:2 in nuclease-free water into two new 96-well plates. Bisulfite converted DNA from these four 96-well plates then transferred into a 384-well plate for real-time PCR analysis, as previously [7,17,23]. The annealing temperatures for the *FMR1* and *SNRPN* assays were 62°C and 65°C, respectively, ran for 40 cycles. Real-time PCR reactions included 4 µL of bisulfite converted DNA, 3.75 µL of MeltDoctor^TM^ master mix (Thermo Fisher Scientific, California, USA), 1.5 µL of nuclease 0.75 µL forward and reverse *SNRPN* or *FMR1* primers targeting CpG sites within promoter regions of these genes as in previous studies [7,17,23].

The QuantStudio^TM^ 7 Flex Real-Time PCR system (Thermo Fisher Scientific, California, USA), was used to quantify DNA concentration post-bisulfite conversion in four replicates per sample, by measuring the rate of dye incorporation into double-stranded DNA using the relative standard curve method, as previously [7,17]. Dynamic linear range (DLR) was determined for *FMR1* and *SNRPN* assays using serial doubling dilutions of control lymphoblast DNA bisulfite converted by either QIAcube HT or IsoPure system. DLR was determined to be: (i) 0.3–10 ng/μL for QIAcube HT converted DNA for both *FMR1* and *SNRPN* analyses, (ii) 0.16–10 ng/μL for *FMR1* and 0.4–10 ng/μL for *SNRPN* IsoPure-based analyses. Of the four bisulfite converted DNA replicates per NBS or DBS punch, only those within this DLR would progress to the high-resolution melting analysis step in close tube format.

The high-resolution melt (HRM) software module for the QuantStudio^TM^ 7 Flex Real-Time PCR system was then used to: (i) plot fluorescence from *FMR1* and *SNRPN* PCR product separation to single strands between 74 °C and 82 °C, (ii) convert measured fluorescence to aligned fluorescence units (AFU). The optimal temperature for separation of AFU between PCR products from methylated and unmethylated target regions was then determined to be: (i) 78.32°C for *FMR1* analyses of IsoPure converted DNA; (ii) 80.87°C for *SNRPN* analyses of IsoPure converted DNA; (iii) 78.53°C for *FMR1* analyses of QIAcube HT converted DNA; (vi) 80.79°C for *SNRPN* analyses of QIAcube HT converted DNA. The AFU measured at these temperatures would then be converted tomethylation ratio (MR) values, with quality control steps assessed simultaneously for 384 reactions at a time using Q-MAX software (Curve Tomorrow, Victoria, Australia), developed to automate the process [17].

The multiple quality control checks included: (i) DNA concentrations postbisulfite conversion being within the dynamic linear range; (ii) DNA concentration post conversion from real-time PCR and AFU values from HRM for each of the four bisulfite conversions per sample being within 35% of their mean. The HRM data from the two neat conversions and two dilutions per sample that did not satisfy these quality checks were not used for quantitative mDNA analysis by the Q-MAX software if (i) the real-time PCR concentration values were outside DLR but were flagged as outside reference range (ORR) for at least one of the replicates used for MR calculation; and/or (ii) real-time PCR and AFU values from HRM for each of the bisulfite conversion replicates were greater than 35% of their mean (flagged by Q-MAX to be repeated). The HRM data from the two neat conversions and two dilutions per sample that did not satisfy these quality checks were discarded from the quantitative mDNA analysis by the Q-MAX software if these did not meet all quality control checks for the minimum of two technical replicates, other than those related to an ORR flag. These were given ND2 flags by the Q-MAX software and were not used for the final aggregate MR calculation from the two neat conversions and two dilutions per sample. Samples with ORR andND2 flags were considered as reaction failures.

### 2.4. Data analysis

Analytical sensitivity for *FMR1* and *SNRPN* analyses was defined as the ability of an assay to differentiate the smallest amount of mDNA from the next smallest amount mDNA on the background of a sample with unmethylated DNA for the target promoter. For *FMR1* analysis, this was DNA from a male affected with FXS with a hypermethylated FM allele on the background of DNA from a neurotypical control male with an unmethylated *FMR1* promoter. For *SNRPN* analysis, this was DNA from an individual with PWS on the background of DNA from an individual with AS and unmethylated *SNRPN* promoter. Diagnostic sensitivity for *FMR1* and *SNRPN* analyses was defined as the ability to correctly identify true positives, being individuals with FXS full mutation or individual chromosome 15 imprinting disorders, respectively. Sensitivity was calculated as the number of true positives over the sum of the number of true positives and the number of false negatives (individuals with negative results but positive for the condition tested) times 100. Specificity for *FMR1* and *SNRPN* analyses was defined as the ability to correctly identify true negatives, being individuals without a FM and FXS or individual chromosome 15 imprinting disorders, respectively. Specificity was calculated as the number of true negatives over the sum of the number of true negatives and the number of false positives (individuals with positive results but negative for the condition tested) times 100.

To assess MS-QMA MR ability to classify the positive and negative cases we used ROC curve analysis. The positive groups were considered as DBS from individuals with diagnosis of FXS or one of the chromosome 15 imprinting disorders confirmed by standard-of-care diagnostic testing. Given that FXS is an X-linked condition, *FMR1* MR analyses were conducted separately for males and females. Area under the ROC curve (AUC) was computed using predicted probabilities from logistic regression, which was used as a summary measure of diagnostic accuracy, and the Youden Index was used to determine the optimal threshold (cutoff point) for MS-QMA analysis for both *FMR1* and *SNRPN* assays [24]. The relationships between MS-QMA MR values from IsoPure and QIAcube HT-based conversion were assessed using simple linear regression analysis. Failure rates were presented as a % of reactions flagged as ND2 and ORR by Q-MAX software of the total number of samples tested. All analyses were conducted using RMS, DiagnosisMed packages, and the publicly available R statistical computing package [25].

## 3. Results

### 3.1. Analytical and diagnostic sensitivity and specificity

We used DNA from lymphoblast cell lines to determine the analytical sensitivity of MS-QMA analyses using both QIAcube HT and IsoPure systems. *FMR1* mDNA analyses were performed on DNA from a neurotypical male control with an unmethylated *FMR1* promoter and CGG size in the normal range spiked at different ratios with DNA from a male affected with FXS and hypermethylated *FMR1* promoter and an FM allele (Figure S3 A and B). *SNRPN* mDNA analyses were performed on DNA from an individual affected with AS and unmethylated *SNRPN* promoter spiked at different ratios with DNA from an individual affected with PWS and hypermethylated *SNRPN* promoter (Figure S3 C and D). Both *FMR1* and *SNRPN* analyses of spiked samples showed linear relationship between the expected versus observed MR values using DNA converted by QIAcube HT and IsoPure systems. Analyses using both QIAcube HT and IsoPure systems showed the same analytical sensitivity of: (i) 0.1 MR or 10% methylation of the *FMR1* promoter; (ii) 0.05 MR or 5% methylation of the *SNRPN* promoter. However, the IsoPure system showed considerably lower intra-run variation than analyses using QIAcub HT especially for analyses on samples with MR of 0.5 or 50% methylation for both *FMR1* and *SNRPN* assays.

**Figure 2.**
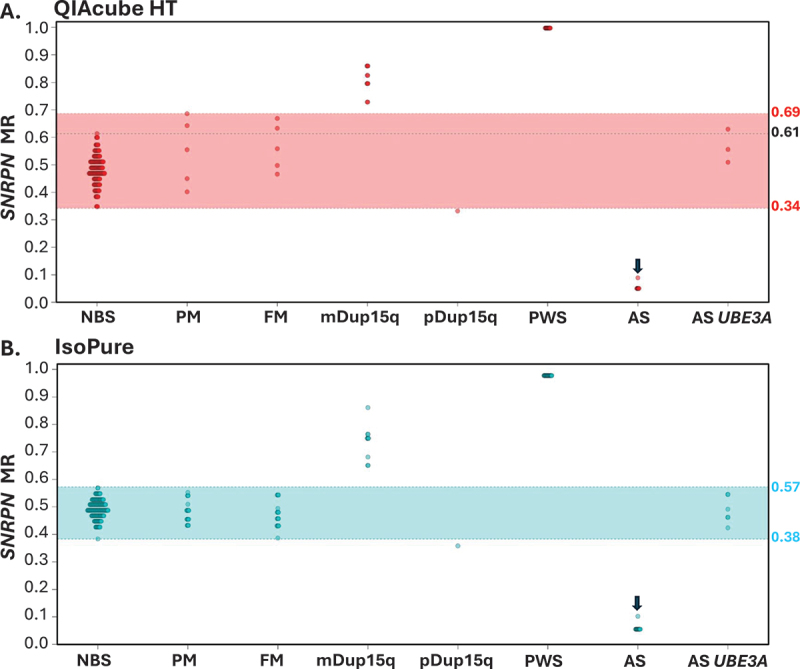
Intergroup comparisons between automated bisulfite conversions using QIAcube HT and IsoPure systems for *SNRPN* methylation analyses on the same archival dried blood spot (DBS, *n* = 90) and freshly made newborn blood spot (NBS, *n* = 184) samples. (A) QIAcube HT and (B) IsoPure bisulfite conversion methods were applied to NBS samples from infants recruited from the general population for de-identified research and DBS samples from individuals confirmed by standard-of-care testing with a chromosome 15 imprinting (C15) disorders (Prader-Willi [PWS], Angelman [AS], maternal Dup15q [mDup15q] or paternal Dup15q [pDUp15q] syndrome), *FMR1* premutation (PM), or with a full mutation (FM) affected with fragile X syndrome. Shaded regions (red for QIAcube HT; blue for IsoPure) indicate the negative control reference ranges for the respective methods. The black line represents the highest MR value observed in NBS samples for each method. The arrow in both (A) and (B) highlights the same individual with a mosaic form of AS. Sample size differences reflect reaction failures flagged by Q-MAX software as either ORR or ND2.

**Figure 3.**
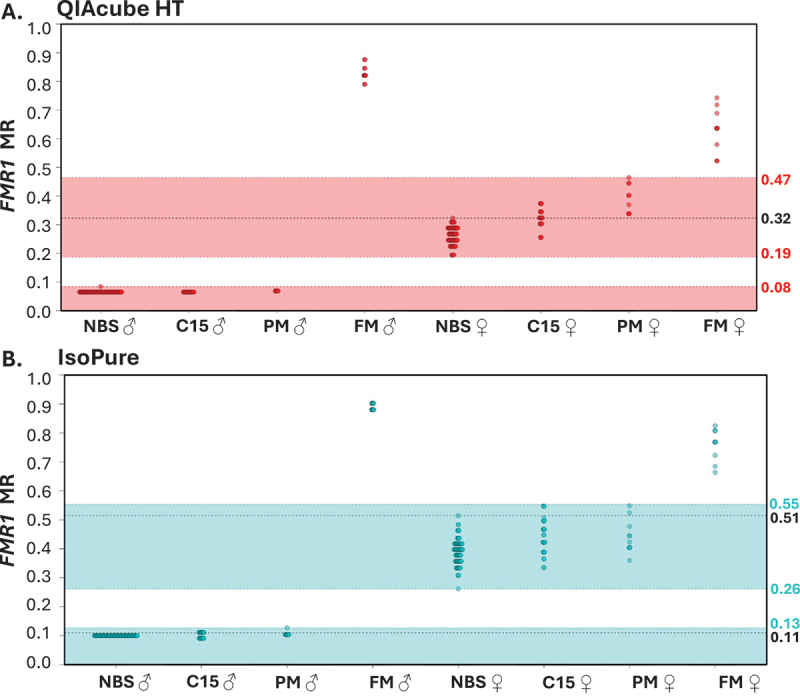
Intergroup comparisons between automated bisulfite conversions using QIAcube HT and IsoPure systems for *FMR1* methylation analyses on the same archival dried blood spot (DBS, *n* = 90) and freshly made newborn blood spot (NBS, *n* = 184) samples. (A) QIAcube HT and (B) IsoPure bisulfite conversion methods were applied to NBS samples from infants recruited from the general population for de-identified research (NBS) and DBS samples from individuals confirmed by standard-of-care testing with a chromosome 15 imprinting (C15) disorders (Prader-Willi [PWS], Angelman [AS], maternal Dup15q [mDup15q] or paternal Dup15q [pDUp15q] syndrome), *FMR1* premutation (PM), or with full mutation (FM) affected with fragile X syndrome. Shaded regions (red for QIAcube HT; blue for IsoPure) indicate the negative control reference ranges for the respective methods. The black lines represent highest MR values observed in male and female NBS samples for each method. Sample size differences reflect reaction failures flagged by Q-MAX software as either ORR or ND2.

For assessments of diagnostic sensitivity and specificity, positive cases were considered as cases with MR values outside the mDNA range observed for blood spots from individuals negative for the conditions screened or the negative control reference range. These were from NBS controls and individuals with conditions and alleles not associated with abnormal mDNA at the target loci (for *FMR1*: PWS, AS, Dup15q and a PM allele; for *SNRPN*: a PM allele, a FM alleles, , AS with *UBE3A* sequence mutation). MS-QMA analysis of *FMR1* and *SNRPN* promoters using both IsoPure and QIAcube HT bisulfite conversion methods demonstrated 100% diagnostic sensitivity and specificity for all screened conditions, excluding AS cases caused by *UBE3A* sequence mutations (Figures 2 and 3). AS cases caused by *UBE3A* sequence mutations were excluded from those expected to be identified by *SNRPN* mDNA testing, as they have previously been shown to exhibit normal *SNRPN* mDNA levels appraoching 0.5 MR [7]. Similarly, 100% diagnostic sensitivity and specificity were observed when the optimal reference ranges for MS-QMA analysis of *FMR1* and *SNRPN* promoers were established using the Youden Index, across all conditions screened for both the IsoPure and QIAcube HT bisulfite conversion systems (Tables 1 and 2) [24].

**Table 1.** Sensitivity and specificity for MS-QMA *SNRPN* methylation analysis in newborn and dried blood spots for chromosome 15 imprinting disorders using area under the curve (AUC) analysis for bisulfite conversion with QIAcube HT and IsoPure systems.

Variable	AUC	SE	95% CI	Optimal cutoff value (Youden index)	Sensitivity	Specificity
**QIAcube HT system**						
mDup15q minimum cutoff	1.000	0.000	1.000–1.000	0.6862	1.000	1.000
mDup15q maximum cutoff	1.000	0.000	1.000–1.000	0.8678	1.000	1.000
PWS	1.000	0.000	1.000–1.000	0.8679	1.000	1.000
AS	1.000	0.000	1.000–1.000	0.0892	1.000	1.000
**IsoPure system**						
mDup15q minimum cutoff	1.000	0.000	1.000–1.000	0.5725	1.000	1.000
mDup15q maximum cutoff	1.000	0.000	1.000–1.000	0.8612	1.000	1.000
PWS	1.000	0.000	1.000–1.000	0.8613	1.000	1.000
AS	1.000	0.000	1.000–1.000	0.1021	1.000	1.000

Note: Standard error (SE), Confidence interval (CI); Maternal chromosome 15 duplication syndrome (mDup15q); Prader-Willi syndrome (PWS); Angelman Syndrome (AS). For mDup15q assessments disease negative group was considered as newborn blood spots (NBS) from infants recruited from general population for de-identified research, dried blood spots (DBS) from individuals with: an *FMR1* permutation (PM), an *FMR1* full mutation (FM), paternal Dup15q (pDup15q), AS due to a deletion, uniparental disomy, or imprinting center defect and AS due to a *UBE3A* sequence mutation and PWS. For PWS assessments, disease negative group was considered as NBS samples from infants recruited from general population for de-identified research, and DBS samples from individuals with: a PM, a FM, pDup15q, AS due to a deletion, uniparental disomy, or imprinting center defect, AS due to a *UBE3A* sequence mutation and mDup15q. For AS assessments, disease negative group was considered as NBS samples from infants recruited from general population for de-identified research and DBS samples from individuals with: a PM, a FM, pDup15q, AS due to a *UBE3A* sequence mutation, PWS and mDup15q.

**Table 2.** Sensitivity and specificity for MS-QMA *FMR1* methylation analysis in newborn and dried blood spots for blood spots from individuals with a full mutation and fragile X syndrome using area under the curve (AUC) analysis for bisulfite conversion with QIAcube HT and IsoPure systems.

Variable	AUC	SE	95% CI	Optimal cutoff MR (Youden index)	Sensitivity	Specificity
**QIAcube HT system**						
Female	1.000	0.000	1.000–1.000	0.4646	1.000	1.000
Male	1.000	0.000	1.000–1.000	0.0842	1.000	1.000
**IsoPure system**						
Female	1.000	0.000	1.000–1.000	0.5535	1.000	1.000
Male	1.000	0.000	1.000–1.000	0.1268	1.000	1.000

Note: Standard error (SE), Confidence interval (CI); Methylation ratio (MR); For sensitivity and specificity assessments of *FMR1* analyses aiming to detect full mutation alleles associated with fragile X syndrome, disease negative group was considered as newborn blood spots (NBS) from infants recruited from general population for de-identified research and dried blood spots (DBS) from individuals with: an *FMR1* premutation, maternal and paternal Dup15q, Angelman and Prader-Willi syndromes.

### 3.2. Intergroup comparisons for *SNRPN* analyses

For chromosome 15 imprinting disorders, *SNRPN* MS-QMA results from 10 matDup15q DBS samples utilizing both IsoPure and QIAcube HT systems showed no overlap with MR values of individuals negative for mDup15q. Using the QIAcube HT method (Figure 2(A)), the matDup15q samples showed distinct MR values compared to 176 NBS from the general population, as well as DBS from 5 individuals with a PM, 5 with a FM affected with FXS, and 24 with other chromosome 15 imprinting disorders. Similarly, the magnetic bead-based IsoPure method (Figure 2(B)) showed mDup15q results clearly separated from 184 NBS from the general population, as well as DBS from 20 individuals with a PM, 19 with FXS, and 39 with other chromosome 15 imprinting disorders.

For PWS DBS samples, 13 samples analyzed using the DNA converted by QIAcube HT system and 19 samples analyzed using DNA converted by the IsoPure system also showed no overlap in MS-QMA *SNRPN* results with the negative control reference groups. The PWS samples’ *SNRPN* MS-QMA results using the QIAcube HT system showed no overlap with values of 176 general population NBS, or with DBS from 5 individuals with a PM, 5 with FXS, and 11 other chromosome 15 imprinting disorders (Figure 2(A)). Similarly, PWS DBS results from DNA converted by the IsoPure system showed no overlap with 184 NBS from the general population, 20 with a PM, 19 with FXS, and 20 with other chromosome 15 imprinting disorders (Figure 2(B)).

Among individuals with AS, 7 DBS samples were analyzed using DNA converted by the QIAcube HT system and 13 DBS were analyzed using DNA converted by the IsoPure system, DNA converted by both methods showing no overlap in MS-QMA *SNRPN* results with the negative control reference groups. For the QIAcube HT system, AS samples had MS-QMA results distinct from 176 NBS from the general population, 5 with a PM, 5 with FXS, and 15 individuals affected with other chromosome 15 imprinting disorders analyzed. Likewise, DNA converted by the IsoPure system showed AS mDNA values clearly separated from 184 general population NBS, 20 with a PM, 19 with FXS, and 26 individuals affected with other chromosome 15 imprinting disorders analyzed (Figure 2(B)). Although the same number of samples was analyzed for conversions by both IsoPure and QIAcube HT systems, differences in the number of MS-QMA MR values were attributed to reaction failures flagged by Q-MAX software as either ORR or ND2.

Of the AS cases tested, there was one individual with mosaicism for *SNRPN* mDNA confirmed by standard of care testing [18]. This case showed the highest *SNRPN* MR among all typical AS cases tested by MS-QMA using both bisulfite conversion methods (Figure 2). As expected, DBS samples from AS individuals caused by *UBE3A* sequence mutations exhibited *SNRPN* MR values within the negative control range for both conversion methods (Figure 2). There was no significant sex-based difference in *SNRPN* MR values among both the NBS from the general population and the known disorder DBS cohort samples using either conversion method (Supplementary Table S2).

The distribution of *SNRPN* MR values for DBS samples differed between the two bisulfite conversion methods. The QIAcube HT method showed *SNRPN* MR values for two of five PM and two of five FM FXS DBS samples above the maximum MR value of the general population NBS samples (Figure 2(A)), whereas for the IsoPure system these samples showed MR values within the general population NBS range (Figure 2(B)).

### 3.3. Intergroup comparisons for *FMR1* analyses

*FMR1* mDNA comparisons were performed separately for males and females as the gene is X-linked with significant differences observed in mDNA between sexes for all groups examined (Supplementary Table S3). For individuals with FXS, 20 DBS samples analyzed using DNA converted by both bisulfite conversion systems showed no overlap in *FMR1* MS-QMA results with the negative control reference groups. DNA converted by the QIAcube HT system showed FXS samples with distinct MS-QMA MR results with no overlap with 18 NBS from the general population, 47 DBS from individuals affected with chromosome 15 imprinting disorders, or 16 DBS from individuals with PM alleles expected to be unmethylated (Figure 3). Similarly, FXS MR results from DNA converted using the IsoPure system showed no overlap with the negative control range for *FMR1*.

As with *SNRPN*, the distribution of *FMR1* MR values for DBS samples was different between the two bisulfite conversion methods. DNA converted using the QIAcube HT system showed 10 out of 47 DBS samples from individuals, with a chromosome 15 imprinting disorder and 9 out of 16 PM DBS samples to have *FMR1* MR values exceeding the maximum observed value in the general population NBS samples (Figure 3(A)). In contrast, DNA converted using the IsoPure only showed 4 out of 49 chromosome 15 imprinting disorder DBS samples and 3 out of 20 PM DBS samples exceeding the control NBS maximum value threshold (Figure 3(B)).

### 3.4. Comparisons of reaction failure rates

We evaluated MS-QMA reaction failure rates for *SNRPN* and *FMR1* mDNA analyses of all 274 samples tested using both bisulfite conversion methods. We also examined failure rates separately for archival DBS from 90 individual with confirmed diagnoses for the conditions screened and freshly made NBS from 184 newborns from the general population. For all 274 blood spots, analyses of *SNRPN* and *FMR1* mDNA reaction failure rates for (i) the QIAcube HT were 19.34% and 2.19%, respectively (Tables 3 and 4), (ii) the IsoPure system were 0.37% and 0.73%, respectively (Tables 3 and 4). The greatest difference in failure rates between the two bisulfite conversion methods was observed in archival DBS samples (Tables 3 and 4).

**Table 3.** Reaction failure rates for *SNRPN* methylation analyses utilizing QIAcube HT and IsoPure systems for bisulfite conversion.

	ORR		ND2		Reaction failure rate (%)
	Number	% of reactions	Number	% of reactions	
**All samples (*n* = 274)**					
QIAcube HT system	9	3.28	44	16.06	19.34
IsoPure system	0	0	1	0.365	0.365
**Archival DBS with confirmed diagnosis of screened conditions (*n* = 90)**					
QIAcube HT system	4	1.46	42	15.33	16.79
IsoPure system	0	0	1	0.365	0.365
**NBS from general population (*n* = 184)**					
QIAcube HT system	5	1.82	2	0.73	2.55
IsoPure system	0	0	0	0	0

Note: DBS = dried blood spots; NBS = newborn blood spots; ORR = outside reference range; ND2 = MR values discarded if a minimum of two technical replicates did not meet quality control checks.

**Table 4.** Reaction failure rates for FMR1 methylation analyses utilizing QIAcube HT and IsoPure systems for bisulfite conversion.

	ORR		ND2		Reaction failure rate (%)
	Number	% of reactions	Number	% of reactions	
**All samples (*n* = 274)**					
QIAcube HT system	2	0.74	5	1.82	2.56
IsoPure system	1	0.37	1	0.37	0.74
**Archival DBS with confirmed diagnosis of screened conditions (*n* = 90)**					
QIAcube HT system	1	0.37	4	1.46	1.82
IsoPure system	0	0	1	0.37	0.37
**NBS from general population (*n* = 184)**					
QIAcube HT system	1	0.37	1	0.37	0.74
IsoPure system	1	0.37	0	0	0.37

Note: DBS = dried blood spots; NBS = newborn blood spots; ORR = outside reference range; ND2 = MR values were discarded if a minimum of two technical replicates did not meet quality control checks.

The NBS samples were further separated into two groups, selected by the 96-well plates with the highest and lowest reaction failure rates from 960 NBS samples screened as part of the EpiGNs program using the QIAcube HT system. For the 92 NBS samples from the plate with the highest failure rates of 1.83% for *SNRPN* and 0.37% for *FMR1* analyses utilizing the QIAcube HT system, analyses using the IsoPure system showed 0% failure rate for both *SNRPN* and 0.37% for *FMR1* analyses (Table 5). For the 92 NBS samples from the plate with the lowest failure rates of 0.73% for *SNRPN* and 0.36% for *FMR1* analyses utilizing the QIAcube HT system, analyses using the IsoPure system showed 0% failure rate for both *SNRPN* and *FMR1* analyses (Table 5).

**Table 5.** Reaction failure rates for *SNRPN* and *FMR1* methylation analyses utilizing QIAcube HT and IsoPure systems for bisulfite conversion of DNA from newborn blood spots.

	Target locus	ORR		ND2		Reaction failure rate (%)
		Number	% of reactions	Number	% of reactions	
**92 NBS samples from the plate with the lowest reaction failure rate**						
QIAcube HT system	*SNRPN*	2	0.73	0	0	0.73
IsoPure system		0	0	0	0	0
QIAcube HT system	*FMR1*	1	0.37	0	0	0.365
IsoPure system		0	0	0	0	0
**92 NBS samples from the plate with the highest reaction failure rate**						
QIAcube HT system	*SNRPN*	3	1.01	2	0.73	1.83
IsoPure system		0	0	0	0	0
QIAcube HT system	*FMR1*	0	0	1	0.365	0.365
IsoPure system		1	0.365	0	0	0.365

Note: ORR = outside reference range; ND2 = MR calues discarded as a minimum of two technical replicates did not meet quality control checks. Newborn blood spot samples were collected from infants recruited from the general population consented for de-identified research. These were either from a plate that showed the lowest or highest reaction failure rate from the first 960 NBS samples screened as part of the EpiGNs program from DNA bisulfite converted using the QIAcube HT system.

### 3.5. Relationships between methylation outputs from analyses using QIAcube HT and IsoPure systems

**Figure 4.**
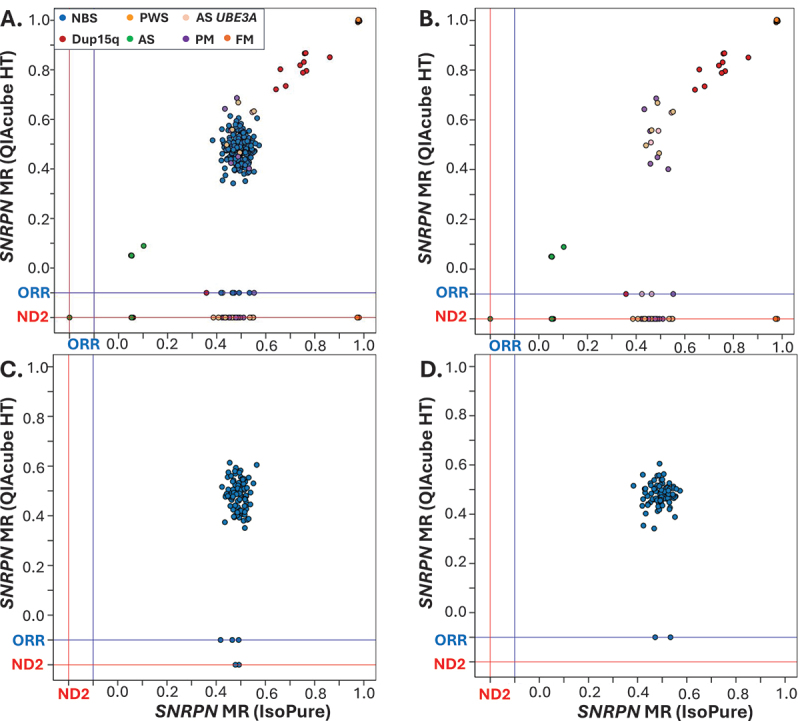
Relationships between QIAcube HT and IsoPure systems for *SNRPN* methylation analyses on the same archival dried blood spot (DBS) and freshly made newborn blood spot (NBS) samples. Outputs from analyses using the IsoPure system are presented on X-axes and QIAcube HT on Y-axes. Comparisons for: (A) all 274 samples analyzed; (B) 90 DBS samples from individuals with conditions screened; (C) 92 NBS samples from the plate with the highest reaction failure rates from 960 NBS samples screened as part of the EpiGNs program using bisulfite converted DNA by the QIAcube HT system; (D) 92 NBS samples from the plate with the lowest reaction failure rates from the first 960 NBS samples screened as part of the EpiGNs program using bisulfite converted DNA by the QIAcube HT system. Note: lines in blue and red respectively depict ‘ORR’ and ‘ND2’ flags by Q-MAX software representing reaction failures in the corresponding system. MR= methylation ratio.

**Figure 5.**
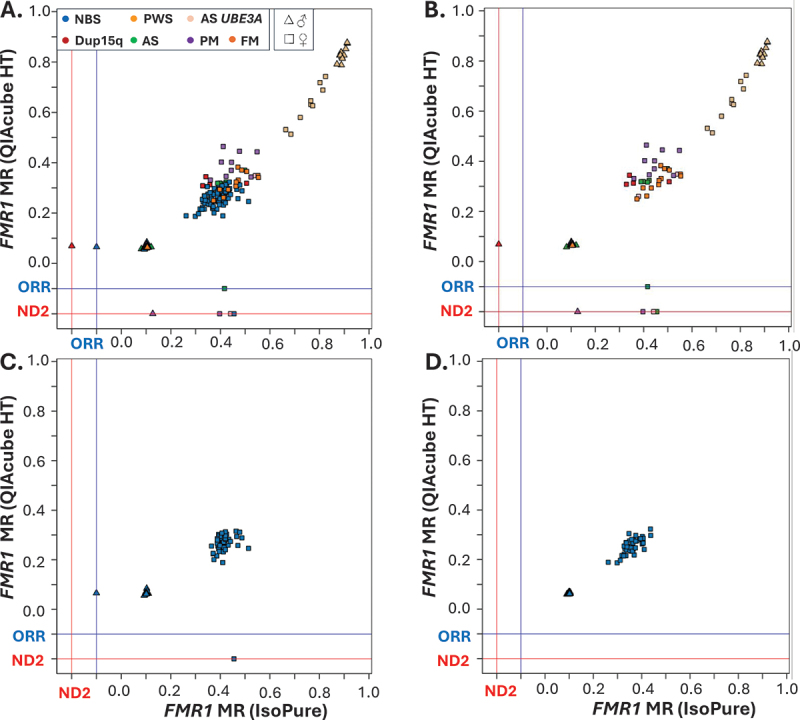
Relationships between QIAcube HT and IsoPure systems for *FMR1* methylation analyses on the same archival dried blood spot (DBS) and freshly made newborn blood spot (NBS) samples. Outputs from analyses using the IsoPure system are presented on X-axes and QIAcube HT on Y-axes). Comparisons for: (A) all 274 samples analyzed; (B) 90 DBS samples from individuals with conditions screened; (C) 92 NBS samples from plate with the highest reaction failure rate from 960 NBS samples screened as part of the EpiGNs program using bisulfite converted DNA by the QIAcube HT system; (D) 92 NBS samples from the plate with the lowest reaction failure rate from 960 NBS samples screened as part of the EpiGNs program using bisulfite converted DNA by the QIAcube HT system. Note: lines in blue and red represent ‘ORR’ and ‘ND2’ flags by Q-MAX software representing reaction failures for each corresponding system. MR = methylation ratio.

Significant relationships were observed for MS-QMA MR values between DNA converted by both QIAcube HT and IsoPure systems for 221 of the total 274 samples analyzed for *SNRPN* and 129 of the total 134 female samples analyzed for *FMR1* mDNA (Figures 4(A) and 5(A); Supplementary Table S4). Significant relationships were also observed between analyses using the QIAcube HT and IsoPure systems for 44 DBS samples from individuals with the conditions screened analyzed for *SNRPN* mDNA and 41archival DBS samples analyzed for *FMR1* mDNA but only from females with the conditions screened (Figures 4(B) and 5(B); Supplementary Table S4). Differences in the number of samples included in these relationships were associated with reaction failures for archival DBS samples from QIAcube HT-based analyses. A significant relationship was observed between analyses using the QIAcube HT and IsoPure systems for female NBS samples from the general population (Figure 5(D); Supplementary Table S4).

## 4. Discussion

### 4.1. Sensitivity, specificity, and distribution of mDNA values

We assessed the analytical sensitivity for *FMR1* and *SNRPN* mDNA analyses of male control or AS DNA with unmethylated target promoters spiked at different ratios with DNA from male with FXS or PWS DNA with hypermethylated target promoters, respectively. The analytical sensitivity was comparable for these mDNA analyses using QIAcube HT and IsoPure systems, between 10% and 5% methylation. However, the IsoPure system showed considerably lower intra-run variability between technical replicates than the QIAcube HT system, especially at methylation levels approaching 50%.

While lower technical variability would be expected to be associated with improved diagnostic sensitivity and specificity, mDNA analyses of *FMR1* and *SNRPN* of blood spots using DNA converted using both systems showed the same diagnostic sensitivity and specificity. These were 100% to differentiate individuals with FXS and chromosome 15 imprinting disorders (excluding AS caused by sequence mutations) from individuals negative for these conditions and newborns from the general population. Moreover, mDNA values from the QIAcube HT and IsoPure systems showed significant relationships with one another from analyses of the same samples. The overall distribution patterns of *FMR1* and *SNRPN* mDNA values were also similar. The *FMR1* mDNA values of DBS from individuals with confirmed diagnosis of FXS were considerably higher than the values on DBS of individuals with a PM, chromosome 15 imprinting disorders or NBS samples from infants recruited from the general population for both sexes. mDNA *SNRPN* values for typical AS samples approached 0 MR, and for typical PWS samples approached 1 MR. *SNRPN* values for matDup15q, pDup15q and mosaic AS were between mDNA ranges for NBS samples from infants recruited from the general population and for DBS from individuals with diagnosis of typical PWS and AS. AS due to *UBE3A* mutation showed *SNRPN* mDNA values that completely overlapped with those of NBS samples from infants recruited from the general population. This was consistent with our previous studies utilizing the QIAcube HT system [7].

However, there were also differences between the systems for the analysis of *SNRPN* mDNA of DBS samples from matDup15q. For these, the IsoPure system-based analyses showed *SNRPN* values to be directly proportional to the number of copies of the duplicated region containing hypermethylated *SNRPN* promoter (Figure S4). This was not the case for analyses using the QIAcube HT system where mDNA values between different matDup15q subtypes overlapped. This suggests that while both systems can be used to identify DBS from individuals with matDup15q, IsoPure system-based analyses are quantitative in the intermediate mDNA range. This is of direct relevance to accurate discrimination of mosaic cases of PWS and AS, as part of newborn screening performed by the EpiGNs program. These cases may show atypical presentation of these conditions and for this reason may not be referred for mDNA testing as early as those with typical presentation of these conditions [18].

### 4.2. mDNA analyses in archival and freshly made blood spots

*FMR1* and *SNRPN* analyses utilizing the QIAcube HT system of archival DBS samples showed greater variability in mDNA than those from freshly made NBS samples from infants ascertained through the general population. This variability resulted in a number of DBS samples from individuals with PM and chromosome 15 imprinting disorders showing *FMR1* mDNA values above the maximum values from the freshly made NBS samples (control population). This phenomenon was likely associated with poor quality of archival DBS samples kept at room temperature for over 10 years, as compared to freshly made NBS samples kept at room temperature for less than 3 months. Similar differences in distribution of *SNRPN* mDNA values between freshly made and archival DBS samples were previously observed for analyses utilizing the QIAcube HT system [7]. In this study for mDNA analyses of the same samples utilizing the IsoPure system, however, did not have these issues. *FMR1* and *SNRPN* mDNA results from archival DBS samples from individuals negative for the conditions screened overlapped with mDNA values from freshly made NBS samples of newborns ascertained through the general population, suggesting that IsoPure system-based analyses are not as sensitive to sample quality issues. This was in line with our comparisons of reaction failure rates between the systems.

### 4.3. Comparisons of reaction failure rates

For the combined set of samples analyzed in this study, reaction failure rates were considerably higher for the QIAcube HT system with 19.34% for *SNRPN* and *2.55%* for *FMR1*, as compared to 0.365% for *SNRPN* and 0.73% for *FMR1* for IsoPure system-based analyses. The considerably higher failure rate for the combined group was over-represented by the failure rate for archival DBS samples.

For both the freshly made NBS samples on NBS plates selected with the lowest and highest reaction failure rates from 960 NBS samples screened as part of the EpiGNs program using the QIAcube HT system, IsoPure-based analyses also showed superior performance, with failure rates approaching 0 for both *FMR1* and *SNRPN* assays. Taken together, these comparisons suggest that IsoPure system-based analyses of mDNA outperformed QIAcube HT-based analyses on both archival and freshly made blood spots.

### 4.4. Strengths and limitations

Key strengths of this study are use of: (i) two sequential punches from blood spots of the same individuals for comparisons between QIAcube HT and IsoPure-based mDNA analyses; (ii) assessments of performance on two distinct loci and for four distinct rare disorders; (iii) relatively large group of positive controls from individuals confirmed to have the conditions screened, considering these are rare disorders; (iv) quality matched archival DBS samples from individuals with chromosome 15 imprinting disorders, and PM, as negative controls for *FMR1* analyses aiming to identify DBS from individuals with FXS; (v) quality matched archival DBS samples from individuals with FXS disorders, and PM, as negative controls for *SNRPN* analyses aiming to detected DBS from individuals with chromosome 15 imprinting disorders; (vi) Q-MAX software to enable quality control assessment of DNA concentration and technical variability post-bisulfite conversion in closed-tube format.

Despite these strengths, the study has a number of limitations which include: (i) use of a relatively small set of NBS samples from the general population; (ii) bias of ascertainment for individuals with the conditions screened. Recruitment of these individuals diagnosed by standard-of-care testing may not represent individuals affected with these conditions in the general population, especially in relation to individuals with mosaicism who may not have typical presentation for these conditions.

Direct mDNA analysis is becoming increasingly accessible with growing availability and affordability of long-read sequencing technologies, such as Oxford Nanopore and PacBio’s single molecule, real-time (SMRT) sequencing. A key advantage these approaches have over the methods used in this study is that they do not require PCR amplification and bisulfite conversion. Additionally, they can sequence complex, repetitive regions – such as CG-rich repeats – that are often challenging to analyze using indirect mDNA analyses. However, despite their potential, these direct mDNA analysis technologies have not yet been adopted into routine clinical practice. These approaches typically require large amounts of high-quality DNA to avoid PCR enrichment steps, and their current cost remains prohibitively high for widespread use in population-scale screening or routine diagnostic testing.

In contrast, MS-QMA remains a cost-effective and practical first-tier screening method within the EpiGN’s workflow and other applications including diagnostic testing for FXS and chromosome 15 imprinting disorders. The loci targeted within *FMR1* and *SNRPN* promoters by MS-QMA do not contain large CG-rich repeat stretches that may cause issues for mDNA analysis. Moreover, their use has been extensively validated on tens of thousands of samples from neurotypical controls and individuals affected with conditions screened (reviewed in [20]). This is not the case for long-read sequencing technologies. Moreover, MS-QMA is compatible with mDNA analysis of a single 3.2 mm NBS punch, making it well suited for possible newborn screening and other high-throughput applications. Use of such limited material, which may be of poor quality and highly fragmented may pose a significant challenge for methylation analysis using long-read sequencing-based methods that do not use PCR amplification.

## 5. Conclusion

This is the first study to assess the performance of different high-throughput automated bisulfite conversion systems for the analysis of *FMR1* and *SNRPN* mDNA. This is also the first report to evaluate DNA methylation analysis using the magnetic bead-based IsoPure system. The study used QIAcube HT and IsoPure systems for bisulfite conversion on the same blood spots, with mDNA assessed using MS-QMA on a limited material of only a single 3.2 mm blood spot punch per individual. Both systems showed a comparable analytical sensitivity of 5% to 10% methylation, with a diagnostic sensitivity and specificity of 100% for FXS and chromosome 15 imprinting disorders. However, analyses using the IsoPure system showed lower reaction failure rates, especially for archival DBA samples of poor quality, more precise quantification of mDNA in the intermediate range of values associated with mosaicism, lower intra-run variability in mDNA values for technical replicates, and lower variability in mDNA values for DBS samples from individuals for the conditions screened and NBS samples from the general population. We, thus, conclude that IsoPure-based analysis has superior performance for screening and diagnostic testing applications for FXS and chromosome 15 imprinting disorders, and potentially other disorders where there is a need for high-throughput mDNA analysis on limited materials of sub-optimal quality.

## Article highlights

- Bisulfite converted DNA is widely used for DNA methylation analysis in gene regulation studies, disease screening, diagnosis, and monitoring, as well as personalized medicine.
- This study examined the performance of DNA methylation screening assays on DNA processed by two high-throughput automated bisulfite conversion protocols using column-based QIAcube HT and magnetic bead-based IsoPure systems.
- *FMR1* and *SNRPN* promoter methylation was assessed using methylation-specific quantitative melt analysis (MS-QMA).
- Assay performance was tested on a single 3.2 mm blood spot punch per individual from the same blood spots of 184 infants consented for de-identified research and 20 individuals affected with fragile X syndrome (FXS) and 50 with chromosome 15 imprinting disorders.
- DNA methylation analyses with both bisulfite conversion systems showed comparable analytical sensitivity of 5% to 10% methylation, with a diagnostic sensitivity and specificity of 100% for FXS and chromosome 15 imprinting disorders.
- Analyses using the IsoPure system showed lower reaction failure rates, especially for archival dried blood spot samples of poor quality, more precise quantification, less variability of DNA methylation values, and lower intra-run variability for technical replicates.
- The study concludes that the IsoPure-based analysis has superior performance for screening and diagnostic testing applications for FXS and chromosome 15 imprinting disorders.
- The study findings have potential implications for other disorders where there is a need for high-throughput methylation analyses on limited materials of sub-optimal quality.

## Supplementary Material

Supplemental Material

## Data Availability

The data that support the findings of this study are available on request from the corresponding author. The data are not publicly available due to privacy or ethical restrictions.
