# Supplementary material for: High-throughput assessment of FMR1 and SNRPN methylation-based newborn screening using IsoPure and QIAcube HT systems
Source: Epigenomics. 2025 Aug 13;17(13):851–63. doi: 10.1080/17501911.2025.2544530 (PMC12369608; doi:10.1080/17501911.2025.2544530)
Supplement: Supplemental Material [file IEPI_A_2544530_SM0518.zip › suppl_data/Figure S4.pptx]

## Slide 1
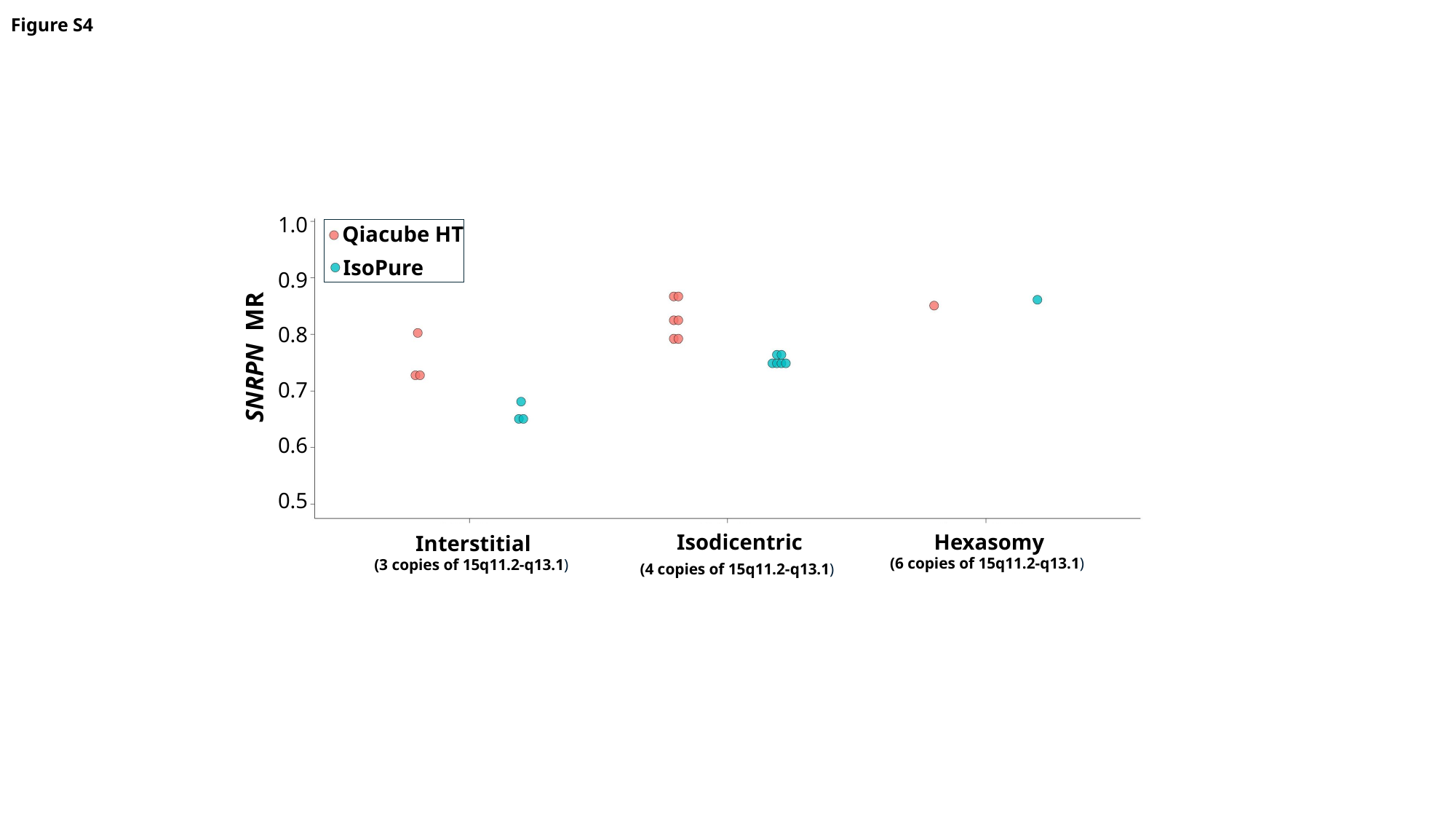

Figure S4
1.0
0.9
0.8
0.7
0.6
0.5
Qiacube HT
IsoPure
SNRPN MR
Isodicentric
(4 copies of 15q11.2-q13.1)
Hexasomy
(6 copies of 15q11.2-q13.1)
Interstitial
(3 copies of 15q11.2-q13.1)
