# Supplementary material for: High-throughput assessment of FMR1 and SNRPN methylation-based newborn screening using IsoPure and QIAcube HT systems
Source: Epigenomics. 2025 Aug 13;17(13):851–63. doi: 10.1080/17501911.2025.2544530 (PMC12369608; doi:10.1080/17501911.2025.2544530)
Supplement: Supplemental Material [file IEPI_A_2544530_SM0518.zip › suppl_data/Supplementary Table S1.docx]

**Supplementary Table S1.** Cohort characteristics.

| **Group** | **Male (N)** | **Female (N)** |
| --- | --- | --- |
| General population (lowest failure rate NBS plate) | 50 | 42 |
| General population (highest failure rate NBS plate) | 45 | 47 |
| Angelman syndrome (DEL) | 4 | 4 |
| Angelman syndrome (UPD) | 3 | 1 |
| Angelman syndrome (ICD) | 1 | 0 |
| Angelman syndrome (Mosaic) | 1 | 0 |
| Angelman syndrome (*UBE3A* sequence mutation) | 3 | 3 |
| Paternal Dup15q syndrome | 1 | 0 |
| Maternal Dup15q syndrome | 6 | 4 |
| Prader-Willi syndrome (DEL) | 2 | 6 |
| Prader-Willi syndrome (UPD) | 4 | 4 |
| Prader-Willi syndrome (ICD) | 1 | 2 |
| *FMR1* premutation | 10 | 10 |
| Fragile X syndrome (FM only) | 9 | 11 |
| Maternal interstitial Dup15q syndrome | 2 | 1 |
| Maternal isodicentric Dup15q syndrome | 4 | 2 |
| Maternal hexasomy Dup15q syndrome | 0 | 1 |
| Paternal Dup15q syndrome | 1 | 0 |

Note: Newborn blood spot (NBS) collected from infants from the general population were consented for de-identified research. These were either from a plate that showed the lowest or highest reaction failure rate from the first 960 NBS samples screened as part of the EpiGNs program from DNA bisulfite converted using the QIAcube HT system. All other samples included were from archival dried blood spots stored at room temperature for over 11 years, made from venous blood of individuals with confirmed diagnosis for the conditions screened. FM = *FMR1* full mutation*;* DEL = Deletion; UPD = Uniparental disomy; ICD = imprinting center defect.
