# Supplementary material for: High-throughput assessment of FMR1 and SNRPN methylation-based newborn screening using IsoPure and QIAcube HT systems
Source: Epigenomics. 2025 Aug 13;17(13):851–63. doi: 10.1080/17501911.2025.2544530 (PMC12369608; doi:10.1080/17501911.2025.2544530)
Supplement: Supplemental Material [file IEPI_A_2544530_SM0518.zip › suppl_data/Supplementary Table S2.docx]

**Supplementary Table S2.** Comparisons of SNRPN methylation ratio values between sexes analysed using DNA bisulfite converted using QIAcube HT and IsoPure systems.

|  | **Female** | | |  | **Male** | | |  |  |
| --- | --- | --- | --- | --- | --- | --- | --- | --- | --- |
|  | **N** | **Median** | **IQR** |  | **N** | **Median** | **IQR** |  | ***p*-value** |
| **QIAcube HT system** | | | | | | | | | |
| NBS | 86 | 0.479 | 0.049 |  | 90 | 0.483 | 0.068 |  | 0.9340 |
| PM | 5 | 0.555 | 0.193 |  | 0 | NA | NA |  | NA |
| FM | 3 | 0.633 | 0.171 |  | 2 | 0.512 | 0.092 |  | 0.2482 |
| matDup 15q | 4 | 0.792 | 0.062 |  | 6 | 0.825 | 0.064 |  | 0.2864 |
| PWS | 9 | 0.997 | 0.005 |  | 4 | 0.996 | 0.003 |  | 0.0641 |
| AS | 3 | 0.051 | 0.039 |  | 4 | 0.051 | 0.001 |  | 0.1573 |
| AS (*UBE3A*) | 2 | 0.593 | 0.073 |  | 1 | 0.509 | NA |  | NA |
| **IsoPure system** | | | | | | | | | |
| NBS | 89 | 0.496 | 0.049 |  | 95 | 0.490 | 0.048 |  | 0.2733 |
| PM DBS | 10 | 0.485 | 0.053 |  | 10 | 0.458 | 0.035 |  | 0.5453 |
| FM DBS | 8 | 0.469 | 0.029 |  | 11 | 0.463 | 0.100 |  | 0.5633 |
| matDup15q | 4 | 0.759 | 0.097 |  | 6 | 0.748 | 0.099 |  | 0.2864 |
| PWS | 12 | 0.979 | 0.003 |  | 7 | 0.978 | 0.002 |  | 0.0759 |
| AS | 7 | 0.053 | 0.008 |  | 6 | 0.052 | 0.003 |  | 0.3173 |
| AS *UBE3A* | 3 | 0.545 | 0.054 |  | 3 | 0.461 | 0.040 |  | 0.0495 |

Note: *p*-value computed using non-parametric Mann-Whitney test; all *p*-values > 0.05 after adjusting for multiple testing using false discovery rate (FDR). Newborn blood spot (NBS) collected from infants from the general population were consented for de-identified research. All other samples were archival dried blood spot (DBS) samples from individuals with confirmed clinical diagnosis of the conditions screened. NBS = newborn bloodspots; PM = *FMR1* premutation*;* FM = *FMR1* full mutation*;* matDup15q = maternal chromosome 15 duplication syndrome; PWS = Prader-Willi syndrome*;* AS = Angelman syndrome caused by a deletion, a uniparental disomy or imprinting center defect; AS (*UBE3A*) = Angelman syndrome caused by *UBE3A* Sequence mutation.
