# Supplementary material for: High-throughput assessment of FMR1 and SNRPN methylation-based newborn screening using IsoPure and QIAcube HT systems
Source: Epigenomics. 2025 Aug 13;17(13):851–63. doi: 10.1080/17501911.2025.2544530 (PMC12369608; doi:10.1080/17501911.2025.2544530)
Supplement: Supplemental Material [file IEPI_A_2544530_SM0518.zip › suppl_data/Supplementary Table S3.docx]

**Supplementary Table S3.** Comparisons of FMR1 methylation ratio values between sexes analysed using DNA bisulfite converted using QIAcube HT and IsoPure systems.

|  |  | **Female** |  |  |  | **Male** |  |  |  |
| --- | --- | --- | --- | --- | --- | --- | --- | --- | --- |
|  | **N** | **Median** | **IQR** |  | **N** | **Median** | **IQR** |  | ***p*-value** |
| **QIAcube HT system** | | | | | | | | | |
| NBS | 88 | 0.264 | 0.045 |  | 95 | 0.063 | 0.002 |  | <0.0001 |
| C15 disorders | 23 | 0.319 | 0.042 |  | 24 | 0.064 | 0.003 |  | <0.0001 |
| PM | 9 | 0.402 | 0.097 |  | 9 | 0.069 | 0.004 |  | 0.0003 |
| FM | 9 | 0.631 | 0.109 |  | 11 | 0.827 | 0.039 |  | 0.0002 |
| **IsoPure system** | | | | | | | | | |
| NBS | 89 | 0.392 | 0.058 |  | 94 | 0.100 | 0.005 |  | <0.0001 |
| C15 disorders | 26 | 0.436 | 0.089 |  | 23 | 0.101 | 0.005 |  | <0.0001 |
| PM | 10 | 0.434 | 0.073 |  | 10 | 0.103 | 0.004 |  | 0.0002 |
| FM | 9 | 0.765 | 0.080 |  | 11 | 0.890 | 0.022 |  | 0.0002 |

Note: *p*-value computed using non-parametric Mann-Whitney test; all *p*-values > 0.05 after adjusting for multiple testing using false discovery rate (FDR). Newborn blood spot (NBS) collected from infants from the general populationre consented for de-identified research. All other samples were archival dried blood spot (DBS) samples from individuals with confirmed clinical diagnosis of the conditions screened. NBS = newborn bloodspots; PM = *FMR1* premutation*;* FM = *FMR1* full mutation*;* C15 disorders = chromosome 15 imprinting disorders.
