# Supplementary material for: High-throughput assessment of FMR1 and SNRPN methylation-based newborn screening using IsoPure and QIAcube HT systems
Source: Epigenomics. 2025 Aug 13;17(13):851–63. doi: 10.1080/17501911.2025.2544530 (PMC12369608; doi:10.1080/17501911.2025.2544530)
Supplement: Supplemental Material [file IEPI_A_2544530_SM0518.zip › suppl_data/Supplementary Table S4.docx]

**Supplementary Table S4.** Relationships between outputs of MS-QMA analyses of SNRPN and FMR1 methylation utilizing QIAcube HT and IsoPure systems.

|  | Spearman's rank correlation | | |
| --- | --- | --- | --- |
|  | N | Estimate | *p* |
| ***SNRPN*** | | | |
| All Samples | 221 | 0.3218 | <0.0001* |
| DBS samples | 44 | 0.9421 | <0.0001* |
| NBS plate with low failure rate | 90 | 0.0545 | 0.6098 |
| NBS plate with high failure rate | 87 | -0.1056 | 0.3303 |
| ***FMR1* (female only)** | | | |
| All Samples | 129 | 0.6927 | <0.0001* |
| DBS samples | 41 | 0.7472 | <0.0001* |
| NBS plate with low failure rate | 42 | 0.6123 | <0.0001* |
| NBS plate with high failure rate | 46 | 0.4384 | 0.0025* |

Note: all *p*-values < 0.05 after adjusting for multiple testing using false discovery rate (FDR). Newborn blood spot (NBS) samples from infants recruited from the general population consented for de-identified research. These were either from a plate that showed the lowest or highest reaction failure rate from the first 960 NBS samples screened as part of the EpiGNs program from DNA bisulfite converted using the QIAcube HT system. All other samples were archival dried blood spots (DBS) from individuals with confirmed clinical diagnosis of the conditions screened. *SNRPN* analyses presented are for both males and females, while *FMR1* analyses are for females only. This is because there is minimal variation in *FMR1* MR values in males – all approaching 0. NBS = newborn bloodspots.
