# Supplementary material for: High-throughput assessment of FMR1 and SNRPN methylation-based newborn screening using IsoPure and QIAcube HT systems
Source: Epigenomics. 2025 Aug 13;17(13):851–63. doi: 10.1080/17501911.2025.2544530 (PMC12369608; doi:10.1080/17501911.2025.2544530)
Supplement: Supplemental Material [file IEPI_A_2544530_SM0518.zip › suppl_data/Supplementary_notes.docx]

**Supplementary notes**

**Supplementary Note S1.** QIAcube HT bisulfite conversion protocol.

For each conversion, 85µL of Bisulfite solution and 35µL of DNA Protect Buffer were added to 20µL of blood spot lysate, with a total reaction volume of 140µL per well in 96 well plates. The plates were then transferred to a thermal cycler for DNA denaturation at 95°C for 5 minutes, followed by incubation at 60°C for 20 minutes, repeated for two cycles. Converted samples were then transferred into a new 2.2mL 96 well S-block (Qiagen, Hilden, Germany) and 560µL of Buffer BL containing 11µg/mL carrier RNA was added to the converted samples.

The samples were then loaded onto the columns of an EpiTect 96 plate and vacuum filtered at 35kPa for 5 minutes using the QIAcube HT instrument (Qiagen, Hilden, Germany). Two wash steps were then repeated twice with 500µL of Buffer BW added by the QIAcube HT instrument (Qiagen, Germany) to each column of the EpiTect 96 plate and vacuum filtered for 2 minutes at 35kPa. 500µL of buffer BD (desulphonation buffer) was then added to each column and incubated at room temperate for 15 minutes before being vacuum filtered at 35kPa for 2 minutes. The samples were then washed again twice with 500µL Buffer BW and vacuum filtered for 30 seconds (35kPa). 250µL of 96% ethanol was then added to the samples and vacuum filtered at 25kPa for 1 minute, at 55kPa for 1 minute, and then at 35kPa for 9 minutes. The EpiTect 96 plate was then incubated for 15 minutes at room temperature before being transferred to an elution plate. The purified converted DNA was eluted from the EpiTect 96 well plate by adding 75µL of Buffer EB, incubating for a minute at room temperature and vacuum filtering at 70kPa for 5 minutes. 30µL of TopElute Fluid was then loaded onto each column and vacuum filtered for 1 minute at 70kPa. 50 µL of purified, converted DNA were then transferred from the elution plate into a new 96-well plate, sealed and stored at -30°C prior to MS-QMA analysis.

**Supplementary Note S2.**  IsoPure bisulfite conversion protocol.

Each bisulfite conversion reaction consisted of 150µL total volume, comprising of 20µL of blood spot lysate and 130µL of Lightning Conversion Reagent, with DNA denaturation and bisulfite conversion performed at 98°C for 8 minutes, followed by 54°C for 60 minutes. For each sample 135µL of the bisulfite converted DNA was transferred to a new deep-well plate containing 600µL of M-Binding buffer (Zymo Research, California, USA) and 10µL of EZ-Methylation MagPrep Beads per well. DNA was then bound to the Magprep beads using IsoPure system’s magnetic tips, with desulphonation, washing and elution steps of the bisulfite converted DNA using L-Desulphonation, M-Wash Buffer and Elution Buffer respectively. DNA samples were bound to the MagPrep beads and then transferred between reagent plates. The elution plate containing the purified bisulfite converted DNA was then sealed and stored at –30°C prior to MS-QMA analysis.
