# Supplementary figures and images for: High-throughput assessment of FMR1 and SNRPN methylation-based newborn screening using IsoPure and QIAcube HT systems
Source: Epigenomics. 2025 Aug 13;17(13):851–63. doi: 10.1080/17501911.2025.2544530 (PMC12369608; doi:10.1080/17501911.2025.2544530)

## Slide 1
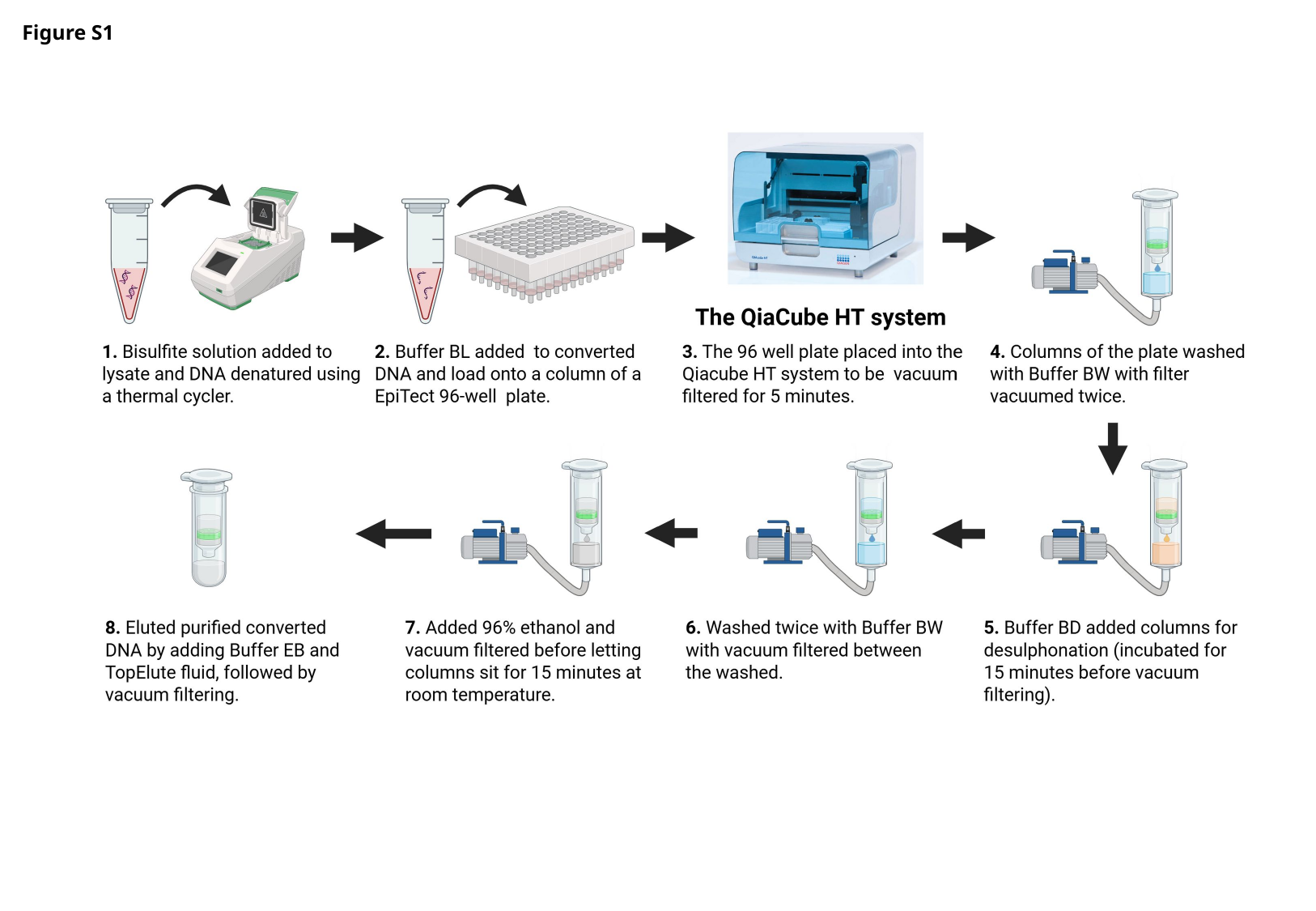

Figure S1

Supplement: Supplemental Material [file IEPI_A_2544530_SM0518.zip › suppl_data/Figure S1.pptx]

## Slide 1
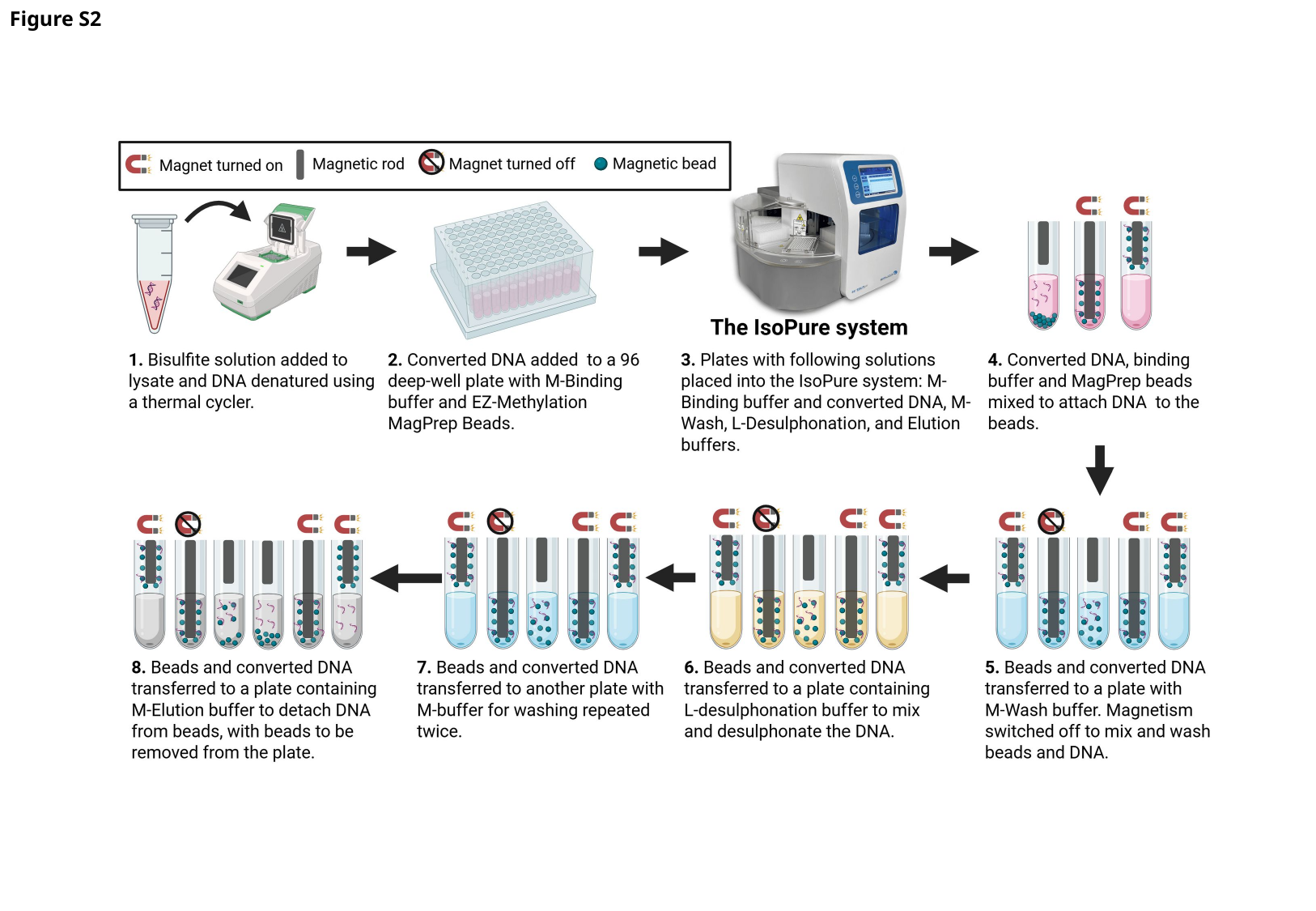

Figure S2

Supplement: Supplemental Material [file IEPI_A_2544530_SM0518.zip › suppl_data/Figure S2.pptx]

Figure S3

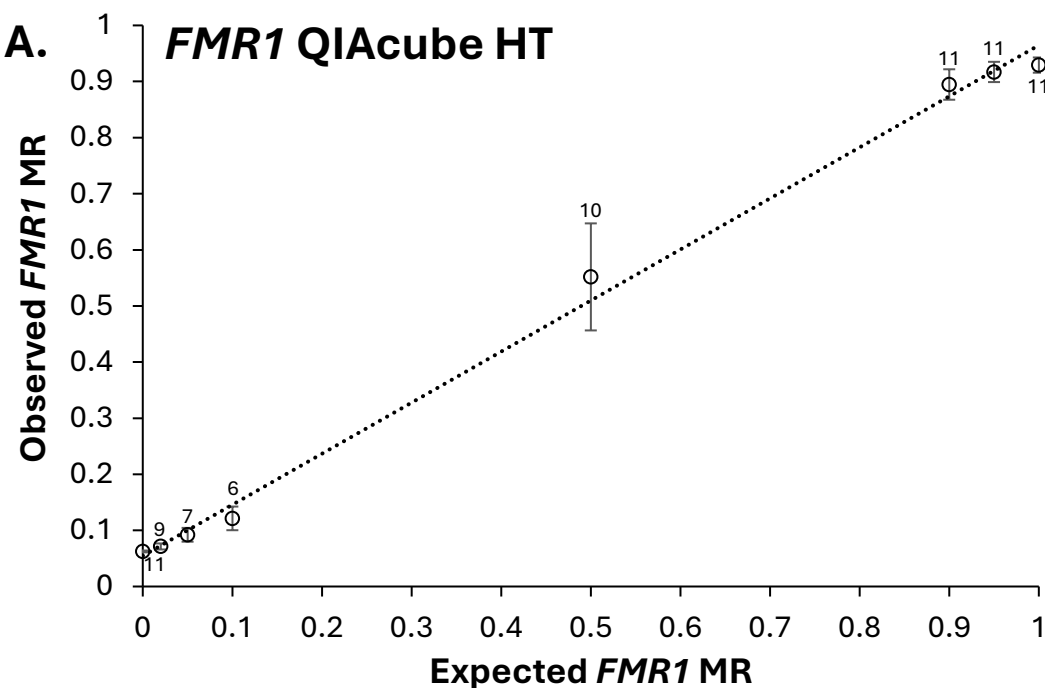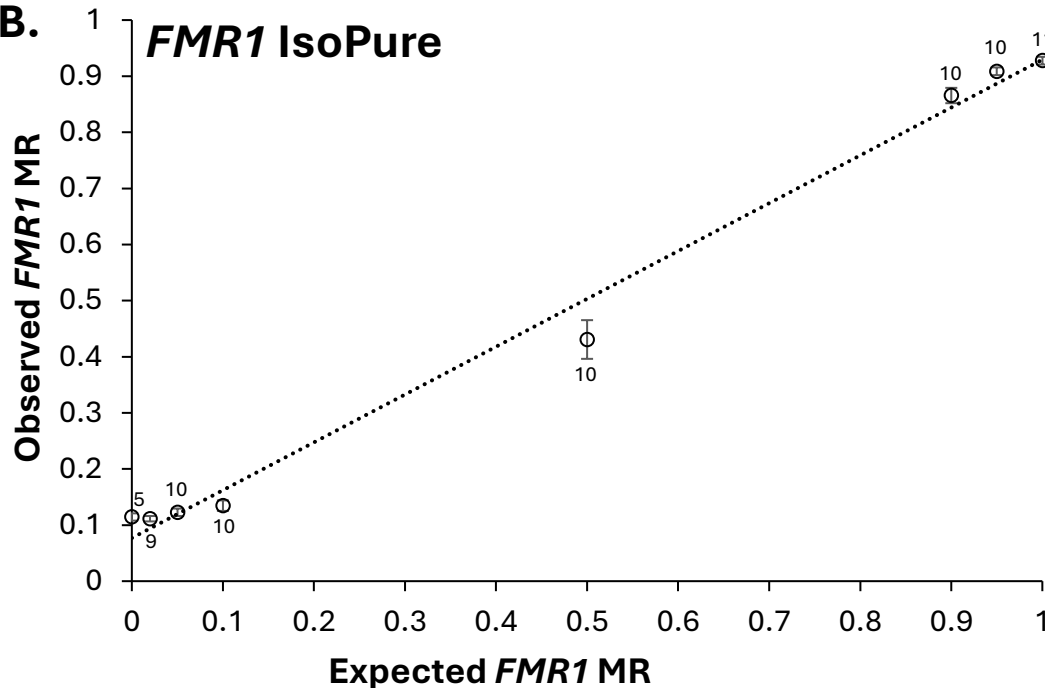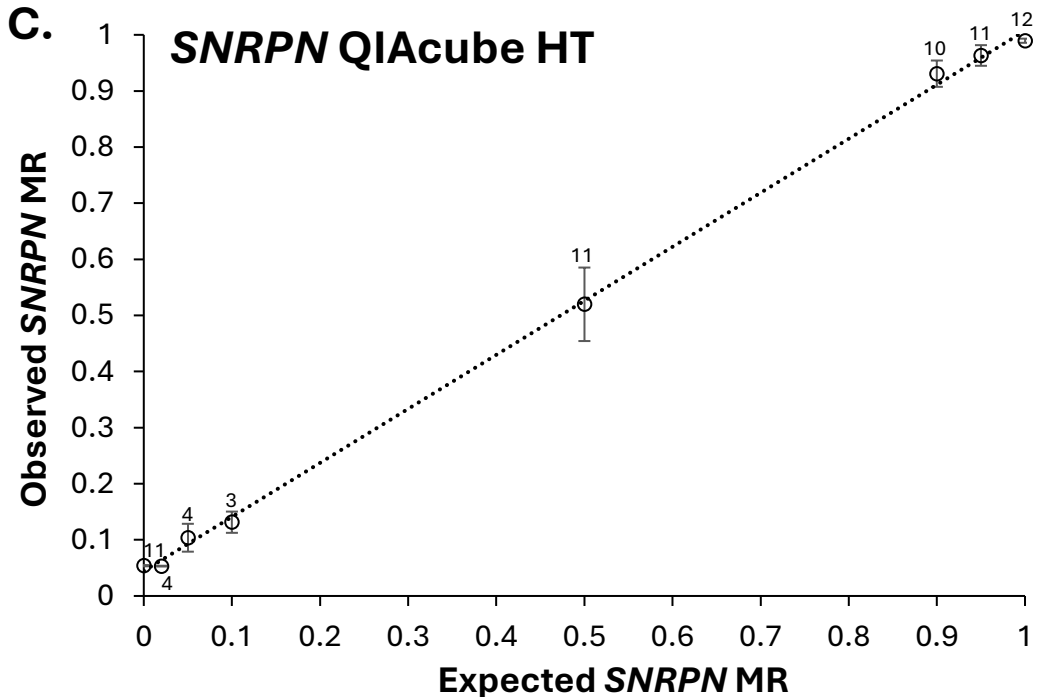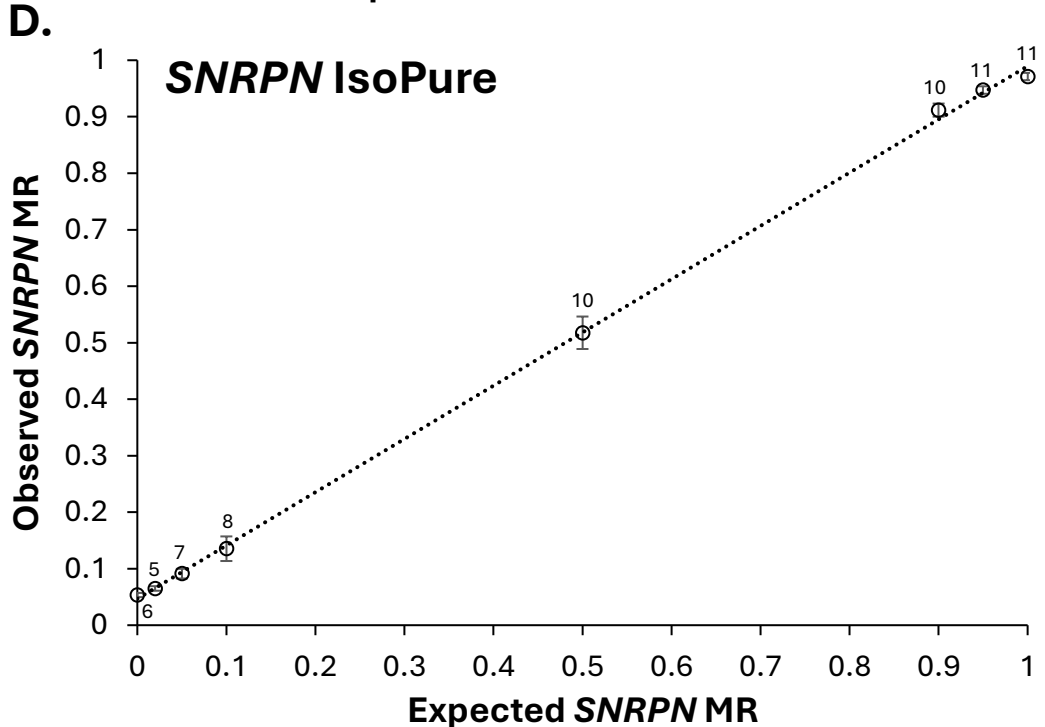

Supplement: Supplemental Material [file IEPI_A_2544530_SM0518.zip › suppl_data/Figure S3.pdf]
